# Estimation of body fat in Pakistani adult: A comparison of equations based upon skinfold thickness measurements

**DOI:** 10.12669/pjms.333.12806

**Published:** 2017

**Authors:** Hafeeza Naz, Kinza Mushtaq, Bilal Azeem Butt, Khadija Irfan Khawaja

**Affiliations:** 1 Dr. Hafeeza Naz, FCPS. Department of Endocrinology and Metabolism, Services Hospital/SIMS Lahore, Pakistan; 2 Dr. Kinza Mushtaq, MBBS. Department of Endocrinology and Metabolism, Services Hospital/SIMS Lahore, Pakistan; 3 Dr. Bilal Azeem Butt, FCPS Department of Endocrinology and Metabolism, Services Hospital/SIMS Lahore, Pakistan; 4 Dr. Khadija Irfan Khawaja, FCPS, Dip Cardiff. Department of Endocrinology and Metabolism, Services Hospital/SIMS Lahore, Pakistan

**Keywords:** Bioelectrical impedance, Correlation, Percentage body fat, Skinfold thickness

## Abstract

**Objective:**

To compare three different body fats estimation equations using skin fold measurements with bioelectrical impedance analysis.

**Methods:**

A total of 130 subjects were included from Department of Endocrinology and Metabolism, Services Hospital, Lahore from 1^st^ April 2016 to 30^th^ Sep. 2016. The triceps, biceps, subscapular, chest, thigh, abdominal, suprailiac skinfold thickness of the subjects was measured with skin-fold calipers (Harpenden) on non-dominant side. The percentage fat mass (%FM) predicted by using each skin-fold-thickness equations namely Durnin & Womersley, Jackson & Pollock and Sloan was compared with %FM measured using a bioelectrical impedance analyzer (BIA).

**Results:**

The mean age of subjects was 48.75±10.7 years, mean BMI was 29.08±6.09 kg/m^2^. The mean %FM calculated by Durnin & Womersley (32.408±0.584), Jackson & Pollock (24.658±0.527), Sloan (20.40±0.545). The %FM by BIA was 38.182±0.529. All three equations showed positive correlation but underestimated %FM as compared to BIA.

**Conclusion:**

All three BF estimation equations underestimate body fat percentage compared to BIA. Among the three, Durnin & Womersley equation shows best positive correlation and hence it can be used for estimation of percentage fat mass as an alternate to BIA.

## INTRODUCTION

The human body is composed of water, muscles, fat, connective tissue and bones.^1^ The body fat is of two types: essential fat and storage fat, also known as adipose tissue.^1^ The essential fat, which is around 8% in men and 12% in women, is crucial for the normal functioning of human body.^1^ While the storage fat or adipose tissue is the non-essential fat and is associated with health risks.^1^

More than 25% of the world’s population is overweight and this percentage is increasing rapidly.^2^ As there are health risks associated with obesity, e.g. ischemic heart disease, Type-2 diabetes, hypertension, efforts have been made to properly quantify body fat in individuals and in different populations.^3^ Most frequently used tool for determination of overweight and obesity status is body mass index (BMI).^4^ In South Asians BMI of > 23.0 kg/m^2^ is considered overweight and above 27·5 kg/m2, is considered obesity.^2^ However, BMI has its limitations as it does not distinguish between body fat and lean body mass.^4^ Moreover, BMI does not correspond to the same degree of fatness because of different body compositions.^5^ Also, some people have a normal BMI but have high levels of body fat, a condition known as “sarcopenic obesity” and some of this fat may be deposited within and around organs (“fat inside,” i.e., obesity based on body fat distribution).^6^ There are certain techniques for accurately assessing total body fat such as underwater weighing (UWW),^7^,^8^ dual energy X-ray absorptiometry (DEXA), and isotope dilution, but they are extremely expensive and are only possible in specialized research centers.^9^,^10^ Bioelectrical impedance is a low cost alternative which is a non-invasive, relatively inexpensive and is portable.^11^,^12^ However it is still limited in utility as it requires specialized equipment and trained operator.^12^

Over the years skin-fold equations have been developed and used for the estimation of percentage of body fat.^13^ The fat estimation by skin-fold is by far the simplest and cost effective method available till now.^14^ These formulae require skinfold thickness measured at multiple sites, to account for the differences in the distribution of bilateral fat at different areas of the body. Harpenden skin-fold calipers are widely accepted as the Gold Standard instrument for skin-fold measurement.^15^

Most of the methods except skin-fold equations require specialized equipment, which is difficult to arrange in resource constrained country like Pakistan. These methods are not practical for use in large epidemiological studies as well as in daily clinical use especially in developing countries like Pakistan. Thus using skin-fold equations is cheaper, more practical and can be used in routine practice to assess body fat, later using this data preventive strategies will be implemented to reduce the morbidity associated with high fat content. However, as the currently available formulae for body fat estimation based upon skin fold thickness are all developed from data based upon western populations it is uncertain if these equations are valid in south east Asian population with the much greater prevalence of adiposity. This study was conducted to compare the body fat estimation by three different skinfold equations namely Jackson & Pollock, Sloan & Durnin and Womersley with BIA.

## METHODS

This study was conducted in Department of Endocrinology and Metabolism, Services Hospital, Lahore from 1^st^ April 2016 to 30^th^ Sep. 2016. We included 130 adult patients (> 18 years of age) from diabetic, endocrine and obesity clinics all of them had diagnosed Diabetes Mellitus according to ADA criteria excluding those patients who had Lipodystrophy or Pacemakers as they are the confounders in estimation of body fat. Written informed consent was taken from all patients (Annex 1). Height and weight of these subjects was measured. Body composition was determined by Bioelectrical Impedance Analysis (BIA) using model Beurer BG64. Skin fold thickness (SFT) was measured by a single trained doctor using Harpenden Caliper from carefully marked sites on triceps, biceps, and subscapular, chest, thigh, and abdomen and suprailiac areas on the non-dominant side. The calipers were calibrated for tension and with a substance of known width prior to testing. Sites were carefully marked and a minimum of two readings at rotating sites were taken. If the two measures at a site differed by more than 3mm, a third measure was taken. The mean of the two closest measures was recorded and used in the calculation of body fat. The estimate of body fat percentage was done in two steps. In the first step, body density was calculated using one of three formulae given below. In the second step, the Siri’s equation was used to calculate the body fat percentage in all three cases. The body fat estimation was also done by bioelectrical impedance analyzer in fasting state as the readings are altered by water intake and activity.

All the demographic data, results from three equations, Siri’s equation and BIA results were collected in a specially designed proforma (annex II). All this data was entered in and analyzed used SPSS version 16 for windows. Mean ± SD was calculated for all quantitative variables like age, BMI and %FM using three body fat estimation equations. BIA was correlated with each of the three fat estimation equations using Pearson correlation.

### Jackson and Pollock

Men: D=1.1125025-0.0013125(x) + 0.0000055(x2) – 0.000244(y)

Women: D=1.089733-0.0009245(x) + 0.0000025(x2) – 0.0000979(y)

Where x=sum of triceps, chest, and subscapular skinfolds (in mm) for men, and the sum of triceps, suprailium, and abdominal skinfolds for women, and y =age in years.

### Sloan equation

Men: D= 1.1043 - (0.001327 x thigh skinfold in mm) - (0.00131 x subscapular skinfold in mm

Women: D= 1.0764 - (0.0008 x iliac crest skinfold in mm) - (0.00088 x tricep skinfold in mm)

### Siri’s Equation

% Body Fat = (495 / Body Density) - 450.

## RESULTS

**Fig.1:**
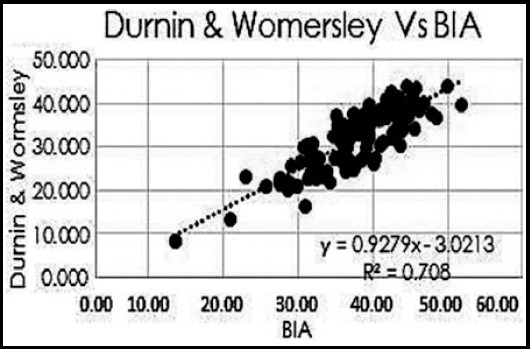
Pearson Correlation between Body Fat percentage by Dunin &Womersley and BIA.

**Fig.2:**
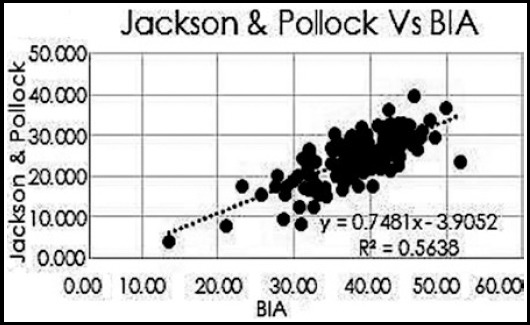
Pearson Correlation between Body fat percentage by Jacksons&Pollock and BIA.

**Fig.3:**
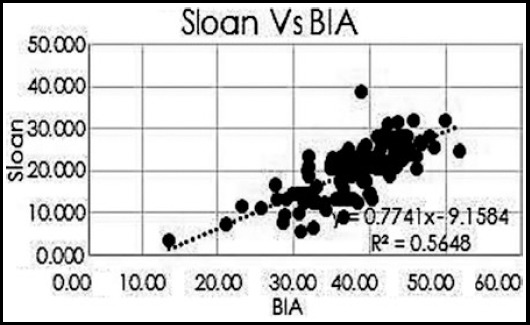
Pearson correlation between Body Fat percentages by Sloan equation and BIA.

The mean age of the participants was 48.75±10.74 years of which 72(55.38%) were males and 58(44.61%) were female. The BMI was 29.08±6.09 kg/m^2^. Overall, mean skin fold measurement at seven different body sites is presented in Table-I. The mean fat deposition is presented in Table-II. It was noted that there was positive correlation between the all the three skinfold thickness equations namely Sloan, Jackson and Pollock (J&P) and Durnin & Womersley (D&W) using Pearson correlation (Table-II), (Fig.1, 2 and 3).

**Table:** Durnin and Womersley:

*Age (Years)*	*Equations For Males*	*Equations For Females*
< 17	D = 1.1533 - (0.0643 × L)	D = 1.1369 - (0.0598 × L)
17-19	D = 1.1620 - (0.0630 × L)	D = 1.1549 - (0.0678 × L)
20-29	D = 1.1631 - (0.0632 × L)	D = 1.1599 - (0.0717 × L)
30-39	D = 1.1422 - (0.0544 × L)	D = 1.1423 - (0.0632 × L)
40 -49	D = 1.1620 - (0.0700 × L)	D = 1.1333 - (0.0612 × L)
> 50	D = 1.1715 - (0.0779 × L)	D = 1.1339 - (0.0645 × L)

D: body density, L: log of sum of 4 skinfolds (biceps, triceps, subscapular and suprailiac).

**Table-I:** Mean skin fold measurement at different body portion.

	*Mean*	*Std. Deviation*
Triceps	16.18	5.508
Biceps	13.30	5.944
Subscapular	21.85	6.657
Thigh	15.12	7.513
Chest	15.89	6.581
Abdominal	25.63	8.019
Suprailiac	17.81	6.579

**Table-II:** Mean fat estimation by equations using Skinfold thickness and BIA.

	*N*	*Minimum*	*Maximum*	*Mean*	*Std. Deviation*	*Std. Error Mean*
BIA	130	13.5	51.8	38.182	6.0392	0.5297
% Body Fat (SLOAN)	130	3.664	39.087	20.40027	6.220976	0.545616
%Body Fat (DURNIN)	130	8.318	44.147	32.40802	6.659969	0.584118
%Body Fat (J&P)	130	4.270	39.806	24.65812	6.016740	0.527703

**Table-III:** Correlation between BIA and other formula of calculation.

*BIA*	*TRP*	*BSP*	*SUB*	*THI*	*CHT*	*ABD*	*SUP*	*BMI*
Pearson Correlation	0.662	0.532	0.574	0.611	0.425	0.480	0.480	0.765
P-value	0.000	0.000	0.000	0.000	0.000	0.000	0.000	0.000

## DISCUSSION

Our study showed positive correlation of all the three equations with bioelectrical impedance analysis (BIA) with Pearson coefficient (R^2^) for Sloan of 0.5648, Jackson and Pollock (J&P) of 0.563 and Durnin & Womersley (D&W) of 0.708.

Estimation of body fat is used in clinical and sports medicine to ensure targeted weight reduction and also to prevent adverse outcomes of adiposity.^15^ Latest scientific studies shows that optimization of fat content of body, results in healthier outcomes than mere weight loss.^16^ The adverse cardio-metabolic profile related to obesity has been documented in many studies.^17^ Adiposity or increased body fat percentage coincides with increased cardiac morbidity and mortality.^17^ Estimation of body composition is thus an essential component of calculating cardiovascular mortality risk. Several methods are utilized for estimation of body fat percentage, of which BIA is comparable to gold standard but needs expertise and specialized machinery.^18^ Skin fold thickness equations are easy to use and do not require special apparatus, hence have a strong practical implication for use in underdeveloped countries like Pakistan.^19^

In our study %FM using BIA was 38.18 ± 6.03, a similar study done in Iran in 2007^20^ showed %FM using BIA of 12.54 ± 6.1 but they collected data only on adolescent boys. Another study done in Canada estimated it as 32.89 ± 8.00^12^ which is comparable to our results. Sloan equation in our study estimated %FM as 20.4 ± 6.2 and Durnin &Womersley equation showed %FM of 32.4±6.65. None of the previous studies have used these two equations for fat estimation.

Our study J & P equation estimated %FM as 24.65 ± 6.02. An Iranian study by Valizadeh A et al.^20^ found %FM using J&P of 6.99 ± 5.3 and concluded that this equation underestimated %FM as compared to BIA, similar results were seen in our study using J&P equation. Another study done in USA Petterson et al.^21^ showed similar results. A meta-analysis done by Fogelholm and Lichtenb et al. in 1997^22^ analysed 54 papers published between 1985 and 1997 and concluded that J & P equation underestimates % FM as compared to BIA but they analyzed only Caucasian population. A large number of studies in various population subgroups have been done and results have been almost similar to ours. To explain J & P equation %FM underestimation in comparison to more reliable and precise methods to measure body composition in many studies. It could be said that while J & P equation was developed there wasn’t any perfect procedure to analyze its validity and nowadays by modern body composition assessment methods its underestimation has been revealed.

Our study has several advantages, firstly there is no need for any special instrument or trained personnel for estimation of skin fold thickness, and caliper used is relatively economical and reasonably accurate and allows zero error removal. Secondly, the equations are easy to compute and have been used in other international studies, so our results can be compared with studies world over. Lastly, to our knowledge our study has the highest number of patients (n=130), no other studies has

### Limitations of the study

This study was done in one tertiary care hospital of Lahore; a multicentric study may show different results. Our study had mostly obese patients, so these results are better applied to such patients only.

## CONCLUSION

All three skinfold equation have a tendency to underestimate body fat percentage when compared to BIA. So we recommend addition of a correction factor in all equations for more accurate estimation of body fat.

***Authors Contribution:***

**HN** conceived, designed and did data collection, statistical analysis & writing of manuscript.

**HN, KM, BAB** did data collection and manuscript editing.

**KIK** did review and final approval of manuscript.
